# Sex differences in response to targeted kyphosis specific exercise and posture training in community-dwelling older adults: a randomized controlled trial

**DOI:** 10.1186/s12891-017-1862-0

**Published:** 2017-12-04

**Authors:** Wendy B. Katzman, Neeta Parimi, Amy Gladin, Eduard A. Poltavskiy, Anne L. Schafer, Roger K. Long, Bo Fan, Shirley S. Wong, Nancy E. Lane

**Affiliations:** 1 University of California, 1500 Owens Street, Suite 400, San Francisco, CA 94158 USA; 2 San Francisco Coordinating Center, San Francisco, CA USA; 3 Kaiser Permanente Northern California, San Francisco, CA USA; 4 University of California, Davis, CA USA; 5 San Francisco Veterans Affairs Health Care System, San Francisco, CA USA

**Keywords:** Kyphosis, Hyperkyphosis, Posture, Aging, Spine, Strengthening exercise, Sex differences

## Abstract

**Background:**

Hyperkyphosis, an excessive anterior curvature in the thoracic spine, is associated with reduced health status in older adults. Hyperkyphosis is highly prevalent, more common in older women than men. There is no standard intervention to reduce age-related hyperkyphosis. Sex differences in response to a kyphosis-specific exercise intervention are not known.

**Methods:**

We conducted a randomized controlled trial of a targeted kyphosis-specific exercise and postural training program on the primary outcome Cobb angle of kyphosis, and investigated whether the magnitude of change differed between men and women. One hundred twelve participants aged ≥60 years with kyphosis ≥40° were enrolled and randomized to exercise or waitlist control, and 101 participants had analyzable baseline and follow-up radiographs for Cobb angle measurements. A group intervention including 10 participants per group was delivered by a physical therapist, 1-h, twice a week for 3-months. Controls were placed on a waitlist for 3 months before receiving a delayed intervention. Primary outcome was change from baseline to 3-months in Cobb angle measured from standing lateral spine radiographs. Secondary outcomes included change over 3-months in kyphometer-measured kyphosis, physical function and quality of life. Groups were combined for analysis after both received the intervention, and sex differences in response to the intervention were tested with ANOVA.

**Results:**

Participants (60 women, 41 men) were 70.0 (SD = 5.7) years old with mean Cobb angle 55.9 (SD = 12.2) degrees at baseline. The active group had higher baseline modified Physical Performance Test scores than control, *p* = 0.03. Men had greater baseline kyphometer-measured kyphosis, *p* = 0.09, and higher bone mineral density (BMD), spine strength, more vertebral fractures and diffuse idiopathic skeletal hyperostosis (DISH) than women, *p* ≤ 0.01. There was no statistically significant difference between groups in change in Cobb at 3-months, *p* = 0.09, however change in kyphometer-measured kyphosis differed by 4.8 (95% CI:-6.8,-2.7) degrees, *p* < 0.001, favoring the active group. There were no differences between men and women in change in either kyphosis measurement after intervention, *p* > 0.1.

**Conclusions:**

A 3-month targeted spine strengthening exercise and posture training program reduced kyphometer-measured, but not radiographic-measured kyphosis. Despite sex differences in baseline kyphosis, BMD, spine strength, fractures and DISH, sex did not affect treatment response.

**Trial registration:**

ClinicalTrials.gov Identifier: NCT01766674.

## Background

Age-related hyperkyphosis, an excessive sagittal plane curvature in the thoracic spine, is associated with reduced physical function and health-related quality of life in older men and women, [1–5] yet there is no standard of care to reduce hyperkyphosis or prevent the progression of kyphosis with age. Older adults with hyperkyphosis have slower walking speed, difficulty climbing stairs and impaired balance which have a negative effect on their satisfaction with life. [1, 6–8] Kyphosis greater than 40 degrees is commonly defined as hyperkyphosis, [1, 2] and once kyphosis progresses beyond 50 degrees, the risk for falls, fractures, [8–11] and mortality increases. [2] In one large cohort study including men and women age 60–70 years old that investigated sex differences in the prevalence of hyperkyphosis, 28% of the women compared to 14% of the men were affected. [9, 12] Underlying musculoskeletal impairments including osteoporosis, vertebral fractures, diffuse idiopathic skeletal hyperostosis (DISH), degenerative disc disease, and spinal extensor muscle weakness and density [13–17] influence the magnitude of kyphosis, and women generally have more of these impairments that may contribute to greater prevalence and progression of kyphosis in women with age. [14–16] Moreover, older men have greater spinal extensor muscle strength [18] and less fatty infiltration into their spinal extensor muscles than women, [19] which may affect the response to an exercise intervention targeted to reduce hyperkyphosis.

Several randomized trials of spinal strengthening interventions have shown improvements in clinical measures of kyphosis [20–23]. Some of these trials included both men and women, although sex differences in treatment effects were not investigated. We previously conducted an uncontrolled pilot study to assess preliminary efficacy of a targeted spine strengthening exercise intervention among 21 older women, and reported a significant improvement in clinically measured kyphosis [24]. Cobb angle was not measured because radiographs were not obtained. Our recent randomized controlled Study of Hyperkyphosis Exercise and Function (SHEAF) trial among 99 older men and women, an average 70.6 (SD = 0.6) years old (range 60–88), demonstrated that spine strengthening exercise and postural training effectively reduced both clinical and radiographic measures of kyphosis in older men and women, [25] but sex differences in response to the intervention were not explored. It is important to investigate whether sex differences in the underlying musculoskeletal impairments that are associated with kyphosis affect the response to a potential treatment of kyphosis. These investigations will inform the medical community about this common and often disabling disorder, help future clinical trial design (e.g., effect size, sex difference), and determine whether a different approach may be warranted for men versus women.

We designed a randomized controlled waitlist trial to determine if a targeted kyphosis specific exercise and posture training program improves the primary outcome of Cobb angle of kyphosis in older community-dwelling men and women, and secondarily to investigate whether the magnitude of change in the outcome of Cobb angle of kyphosis before and after the intervention differs between men and women. Additionally, we included secondary outcome measures of physical function, and health-related quality of life (HRQoL) that are affected by age-related hyperkyphosis.

## Methods

### Participants

Participants were recruited from January 2013 through June 2015 from local senior centers and outpatient medical clinics at 2 large urban medical centers (a university-based center and an integrated managed-care center). Once screened on-line or by telephone, a baseline screening examination was scheduled, written informed consent was obtained and permission from the potential participant’s primary care provider was obtained.

Inclusion criteria were the same as the previously published SHEAF clinical trial, [25] and included proficiency in English, age 60 years or older, kyphosis angle 40 degrees or higher (measured with a kyphometer at the screening visit), ability to walk one block without an assistive device, able to climb one flight of stairs independently, and rise from a chair without the use of one’s arms. Participants were excluded for inability to actively reduce their kyphosis measurement by at least 5 degrees, cognitive impairment on the Mini-Cog, [26] inability to pass safety tests in the screening examination or any disorder or disease likely to prevent or interfere with safe participation in a group-based exercise program.

The study protocol was approved by the University of California San Francisco and Kaiser Permanente Northern California Institutional Review Boards.

### Randomization

**Fig. 1:**
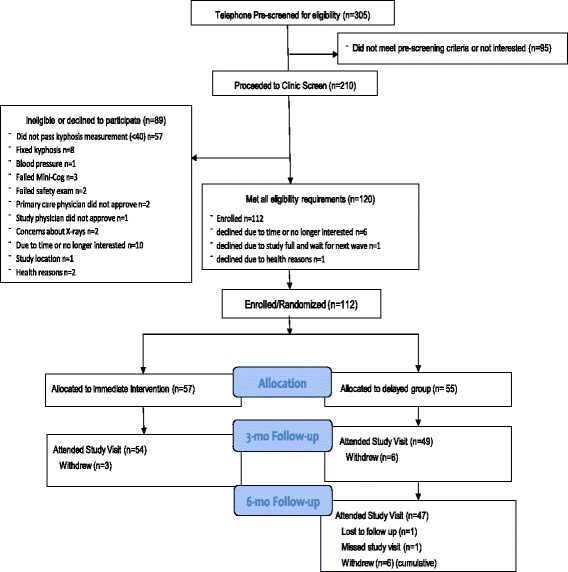
Consort Diagram – Participant recruitment and retention

**Fig. 2:**
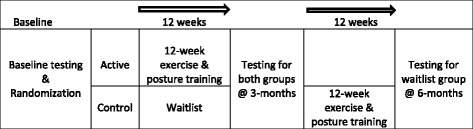
Study flow

The study was enrolled in five waves of 20 participants each (Fig. 1). Following baseline testing, participants were randomized to the active or waitlist control group in randomly permuted blocks of 2 and 4, stratified by age (<75 vs 75+) and sex (male vs female). A random allocation sequence was generated by the study biostatistician, and placed in sealed consecutively numbered envelopes according to age and sex stratum. An envelope with the next available ID number for the appropriate age and sex stratum was opened after completing baseline testing. The active group received the intervention immediately and the waitlist control group received a delayed intervention after the 3-month waitlist (Fig. 2).

### Intervention

Active participants attended a group (*n* = 10) exercise program for 1 h two times per week for 3 months. A licensed physical therapist, assisted by a trained research assistant, led the exercise intervention. The structure of the intervention was the same as previously published studies [24, 25, 27] of a multi-modal group-based kyphosis-specific exercise and posture training program that targeted spinal extensor muscle strength, [14, 23] spinal mobility, [28] and postural alignment [24, 27, 29]. Exercises were progressed in intensity during the study to maintain a Borg Scale intensity of 4–5, based upon 70–80% of perceived exertion, [30] while maintaining good quality movement.

The instructors used postural training [31] and provided verbal, visual and tactile feedback to participants to assist them in learning and maintaining neutral spinal alignment during the exercises. Participants were also given a study manual of pictures depicting neutral spinal alignment during activities of daily living. They were instructed to practice neutral spinal alignment 3 times or more a day outside of study visits, and report compliance with this home program on a weekly basis to the study coordinator.

### Control

Control participants were placed on a waitlist for 3 months and received a delayed intervention after the initial 3 month waitlist. During the waitlist period, the study coordinator contacted them by phone on a monthly basis to assess adverse events. After the 3-month waitlist period, all primary and secondary outcome measurements were repeated prior to receiving the 3-month group exercise intervention. After receiving the group exercise intervention, participants were tested again at 6-months after enrollment.

### Outcome assessments

#### Primary outcome: Change in Cobb angle of kyphosis

**Fig. 3:**
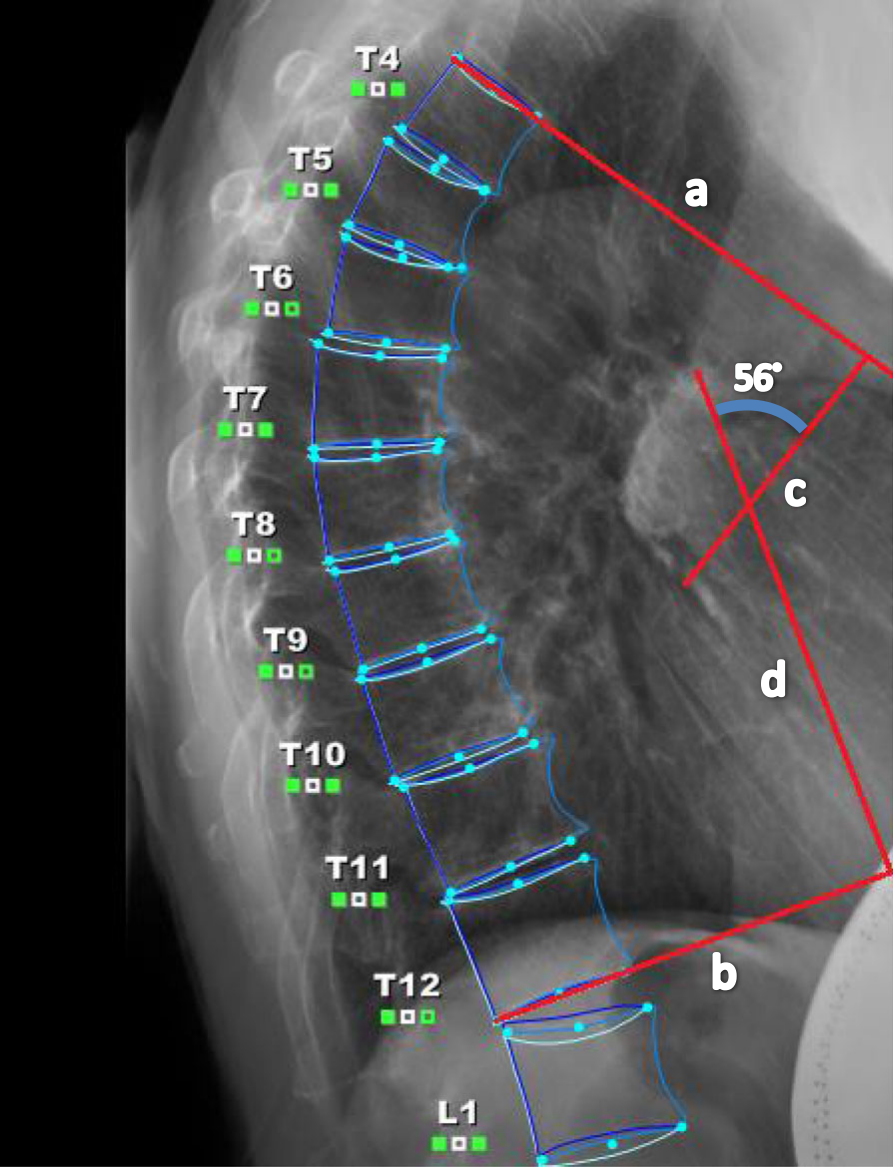
Cobb angle of kyphosis (56 degrees) measured from standing lateral radiograph Line a is drawn from the superior endplate of T4; line b is drawn from the inferior endplate of T12; lines c and d are perpendicular lines drawn from lines a and b. Cobb angle of kyphosis (56 degrees) is where lines c and d intersect

A baseline assessment was conducted before randomization and included all primary and secondary outcome measurements. The primary outcome of change in kyphosis was assessed between baseline and 3 months using the gold standard Cobb angle derived from standing lateral spine radiographs and a standardized protocol for measurement of thoracic kyphosis (the angle formed from intersecting lines drawn from the superior endplate of T4 and the inferior endplate of T12) (Fig. 3) [32]. Test-retest reliability for repeated measurement of Cobb angle from the same radiograph was estimated from 30 baseline radiographs as ICC = 0.90. Standard error of the measurement was previously estimated as 1.4 degrees in a previous study [25].

#### Secondary outcomes: Change in kyphometer-derived kyphosis, physical function, spinal extensor muscle strength and HRQoL

Secondary outcomes included change in kyphometer-derived kyphosis, physical function, spinal extensor muscle strength, and HRQoL, and were assessed between baseline and 3 months in both groups. Clinical measurements were made by a trained staff member at the UCSF Clinical and Translational Science Institute who was blinded to group allocation. The Debrunner kyphometer (Techmedica Inc., Camarillo, CA) was used to obtain an external measurement of kyphosis using the T2/3 spinous process interspace as the superior landmark and T11/T12 spinous process interspace as the inferior landmark using a standard protocol [32]. The modified Physical Performance Test (modified PPT) [33] included 7 timed standardized functional tasks. Gait speed (m/s) was calculated for a timed walk administered over a 4-m marked course [34]. The Timed Up and Go test (TUG) measured the time in seconds to rise from a 41-cm height armchair, walk 3 m, turn and return to a fully seated position in the chair [35]. Timed Loading Standing measured the time in seconds a participant was able to stand while holding a two-pound dumbbell in each hand with the arms at 90 degrees of shoulder flexion and the elbows extended [36]. The Six-minute Walk test measured the distance in meters covered while walking in a long hallway for 6 min [37]. Participants completed the Scoliosis Research Society (SRS-30) instrument, self-image domain, [38] and the PROMIS global health index (with mental and physical health components) and physical function quality of life questionnaires. [39] Isometric spinal extension and flexion muscle strength were assessed using a standardized protocol [24] with the Biodex 3 (Biodex Medical Systems Inc., Shirley, NY) computerized dynamometer and the spine attachment (RSI Systems, Boulder, CO). Peak torque (pounds) normalized to body weight was used to quantify strength.

All primary and secondary outcome measurements were repeated at baseline and 3 months. Those assigned to the waitlist received an additional testing visit, which included all primary and secondary outcome measures at 6-months, after completing the 3-month exercise and training intervention.

#### Other measures

At baseline, we measured height and weight using standard methods, and calculated body mass index (BMI). Bone mineral density was measured using dual-energy X-ray absorptiometery (DXA) (GE Lunar Prodigy), and sex-specific t-scores for the hip and spine were derived. A radiologist (BF) assessed prevalent vertebral fractures in T4 to L4 vertebrae from baseline standing lateral spine radiographs using the Genant semi-quantitative (SQ) method grading fractures 0–3, where 0 = none (normal), 1 = mild, 2 = moderate, and 3 = severe [40]. We defined a prevalent vertebral fracture as SQ ≥2. Another radiologist (LN) evaluated the radiographs for the presence of diffuse idiopathic skeletal hyperostosis (DISH) from T4 to L4 using the Resnick criteria [41].

Adverse events were monitored by the study coordinator, who administered a standardized questionnaire on a weekly basis in the active group and in short monthly phone interviews in the control group. Pain was documented using the visual analog pain scale, [42] and falls and other injuries were documented. Events were recorded as occurring during or outside of a study visit, and as pre-existing or a new event.

### Statistical methods

Baseline characteristics of the active and control groups, and men and women were compared using t- and chi-square tests, as appropriate. First, ANCOVA was used to assess differences in change in the primary and secondary outcomes from baseline to 3-months in the active and control groups while controlling for the pre-treatment outcome. In addition, to determine the difference-in-difference and 95% confidence interval of the pre- and post-treatment changes between the active and control groups, we regressed the within person change in primary and secondary outcomes on treatment group. Then, we combined the active and control groups after both groups received the intervention, and ANOVA was used to test a sex by treatment interaction to evaluate differences in change in the outcomes before and after the intervention in men and women. We planned a modified intention to treat analyses with participants who had readable baseline and follow-up radiographs of sufficient quality to be evaluated to compare change in the primary Cobb angle outcome. Thus we excluded those that had radiographs that could not be evaluated and those who withdrew from the study during the first 2 weeks due to lack of time or interest. *P*-values <0.05 were considered statistically significant. In exploratory analyses, we assessed differences in the treatment effect by the presence of diffuse idiopathic skeletal hyperostosis (DISH) (yes, no) and vertebral fractures (SQ ≥ 2). In these analyses, we tested for interactions with *p*-values <0.1 considered statistically significant. Based upon results from our prior pilot study, we determined that a sample size of 100 participants would have 80% power in 2-sided tests with a type-I error rate of 5%, allowing for within-subject correlation of the baseline and 3-month outcomes, and loss to follow-up of 20% of participants, to detect a clinically meaningful change of 2 degrees (or more) in the primary outcome Cobb angle of kyphosis. Additionally we determined a priori that a sample including 40% men would have 84% power to detect a 2-degree difference between men and women in mean change in the primary outcome. We calculated the minimal detectable difference while using the pooled standard deviation from ANCOVA models, modeling the change after treatment in Cobb angle in active versus controls while controlling for baseline values. Analyses were conducted using SAS 9.4 (SAS Institute, Cary, NC).

## Results

### Subject characteristics and clinical variables

One hundred twelve participants were enrolled and randomized to exercise (*n* = 53) or waitlist control (*n* = 48), however 9 withdrew within the first week due to lack of time or interest, and 2 did not have analyzable baseline radiographs for Cobb angle measurements (Fig. 1). The remaining 101 participants (60 women, 41 men) with 3-month follow-up data for Cobb angle of kyphosis were on average 70.0 (SD = 5.7) years old with baseline Cobb angle 55.9 (SD = 12.2) degrees (Table 1). Mean gait speed in our cohort was 1.3 (SD = 0.3) m/s. Eighty-three percent of the cohort was classified as “not frail” (score 32–36) with the remaining 17% classified as “mild frailty” (score 25–31) according to the modified PPT [33]. Five (9%) participants in the active group versus 12 (25%) in the control group were classified as “mild frailty” on the modified PPT, *p* = 0.04. There were no other significant differences between the active and control groups at baseline, *p* > 0.05. Men had greater kyphometer-derived kyphosis (*p* = 0.09), and greater bone mineral density and spine strength, more vertebral fractures, and DISH (*p* ≤ 0.01) compared to women (Table 2).

**Table 1:** Study participant characteristics at baseline between groups

Variable	Overall	Active	Control	*p*-value
	(*N* = 101^a^)	(*N* = 53^a^)	(*N* = 48^a^)	
	Mean ± SD			
Age (years)	70 ± 5.7	69.4 ± 5.8	70.0 ± 5.7	0.62
Cobb angle of kyphosis (degrees)	55.9 ± 12.2	57.5 ± 13.6	54.2 ± 10.4	0.18
Kyphosis derived from kyphometer (degrees)	52.0 ± 7.4	51.4 ± 7.9	52.7 ± 7.0	0.37
BMI (kg/m^2^)	26.2 ± 4.1	26.0 ± 4.3	26.4 ± 3.9	0.64
Bone mineral density total hip t-score	−0.9 ± 1.1	−1.02 ± 1.0	−0.7 ± 1.2	0.10
Bone mineral density total spine t-score	−0.2 ± 2.2	−0.5 ± 1.8	0.2 ± 2.5	0.09
	N (%)			
Race/ethnicity – Caucasian	94 (93)	50 (94)	44 (92)	0.60
Education				0.85
Some college, vocational or high school	13 (13)	6 (14.5)	7 (15)	
College graduate (BA, BS)	33 (33)	17 (32)	16 (33)	
Professional or graduate degree	55 (54)	30 (54)	25 (52)	
Co-morbidities				0.85
0–1	61 (60)	25 (61)	36 (60)	
2+	40 (40)	16 (39)	24 (40)	
Diffuse idiopathic skeletal hyperostosis (DISH) (yes)	22 (23)	14 (27)	8 (17)	0.24
Vertebral fracture				0.16
None	87 (86)	48 (91)	39 (81)	
Mild	9 (9)	2 (4)	7 (15)	
Severe	5 (5)	3 (6)	2 (4)	

^a^restricted to participants with readable baseline and follow-up radiographs of sufficient quality tthat could be evalauted to compare change in the primary Cobb angle outcome; kg/m^2^ = kilogram/m^2^; SQ = semi-quantitative Genant scoring

**Table 2:** Study participant characteristics at baseline between men and women

Variable	Overall	Men	Women	*p*-value
	(*N* = 101^a^)	(*N* = 41^a^)	(*N* = 60^a^)	
	Mean ± SD			
Age (years)	70 ± 5.7	70 ± 5.5	69 ± 6.0	0.30
Cobb angle of kyphosis (degrees)	55.9 ± 12.2	54.6 ± 12.3	56.7 ± 12.1	0.40
Kyphosis derived from kyphometer (degrees)	52.0 ± 7.4	53.5 ± 7.3	51.0 ± 7.4	0.09
BMI (kg/m^2^)	26.2 ± 4.1	27.2 ± 3.9	25.6 ± 4.1	**0.04**
Bone mineral density total hip t-score	−0.9 ± 1.1	−0.3 ± 1.2	−1.2 ± 0.9	**<0.001**
Bone mineral density total spine t-score	−0.2 ± 2.2	0.9 ± 2.5	−0.9 ± 1.6	**<0.001**
	N (%)			
Race/ethnicity – Caucasian	94 (93)	38 (93)	56 (93)	0.90
Education				0.91
Some college, vocational or high school	13 (12.9)	6 (14.5)	7 (12)	
College graduate (BA, BS)	33 (33)	13 (32)	20 (33)	
Professional or graduate degree	55 (54)	22 (54)	33 (55)	
Co-morbidities				0.84
0–1	61 (60)	25 (61)	36 (60)	
2+	40 (40)	16 (39)	24 (40)	
Diffuse idiopathic skeletal hyperostosis (DISH) (yes)	22 (23)	17 (43)	5 (9)	**<0.001**
Vertebral fracture				**0.01**
None	87 (86)	31 (76)	56 (93)	
Mild	9 (9)	5 (12)	4 (7)	
Severe	5 (5)	5 (12)		

^a^restricted to participants with readable baseline and follow-up radiographs of sufficient quality tthat could be evalauted to compare change in the primary Cobb angle outcome; kg/m^2^ = kilogram/m^2^; SQ = semi-quantitative Genant scoring

The minimal detectable difference in Cobb angle for a two sided test with 80% power was 2.6 (95% CI, 2.29–3.03).

### Intervention adherence

Participants in each group attended an average of 20 (SD = 5) (84%) of the 24 scheduled exercise classes. Overall 78% (range 0–100) completed the daily home program, and 98% (range 50–100) completed the home program at least 3 or more days a week. There was no difference in attendance in men versus women, *p* = 0.74. However, 70% (range 0–100) of the men versus 83% (range 0–100) of the women completed the daily home program, and 95% (range 50–100) of the men versus 99% (75–100) of the women completed the home program at least 3 or more days a week.

### Change in Cobb angle of kyphosis

In the primary outcome of Cobb angle of kyphosis, participants in the intervention group had a 1.4 (95% CI, −2.7 to −0.1) degree decrease in Cobb angle while in the control group Cobb angle increased by 0.3 (95% CI, −1.1 to 1.7) degrees for a difference-in-difference of 1.7 degrees. The difference was not statistically significant at α = 0.05 level, with a *p*-value of 0.09 (Table 3).

**Table 3:** Post intervention differences in outcomes over 3 months between active and control groups

	Within group LS mean difference (pre/post treatment) (95% CI)		^1^Between group difference (95% CI)	
Outcome	Control	Active	^a^DID	^2^ *p*-value
	(N = 48)	(n = 53)		
	Primary Outcome			
Cobb angle kyphosis (degrees)	0.3 (−1.1 to 1.7)	−1.4 (−2.7 to −0.1)	−1.7 (−3.6 to 0.2)	0.09
	Secondary Outcomes			
Kyphometer kyphosis (degrees)	1 (−0.5 to 2.4)	−3.8 (−5.3 to −2.3)	−4.8 (−6.8 to −2.7)	**<0.0001**
Modified PPT (0–36 points)	0.5 (−1.2 to 2.2)	−0.9 (−2.6 to 0.7)	−1.4 (−3.8 to 0.9)	0.23
4-m (meters/s)	−0.03 (−0.09 to 0.03)	−0.03 (−0.09 to 0.02)	0 (−0.08 to 0.08)	0.94
Timed Up and Go (seconds)	0.2 (−0.1 to 0.5)	0 (−0.4 to 0.3)	−0.2 (−0.7 to 0.2)	0.33
Timed Loaded Standing (seconds)	−6.2 (−14.2 to 1.7)	−3.6 (−11.7 to 4.4)	2.6 (−8.5 to 13.7)	0.64
Six Minute Walk Test (meters)	−17.7 (−36.6 to 1.2)	4.4 (−14.6 to 23.3)	22.1 (−4.2 to 48.4)	0.10
SRS 30 Self-esteem (0-5points)	0.1 (0 to 0.2)	0.2 (0.1 to 0.3)	0.1 (−0.1 to 0.3)	0.16
Global health (0–50)	0.6 (−0.5 to 1.7)	0.2 (−0.8 to 1.3)	−0.4 (−1.8 to 1.1)	0.64
Physical function (t-score)	1.5 (−0.3 to 3.3)	1.3 (−0.4 to 3)	−0.2 (−2.6 to 2.2)	0.86
Spinal extension peak torque (% body weight)	1.8 (−3.2 to 6.8)	8 (2.9 to 13)	6.1 (−0.9 to 13.1)	0.08
Spinal flexion peak torque (% body weight)	−0.7 (−2.6 to 1.3)	2 (0.1 to 4)	2.7 (0 to 5.4)	0.05

^a^DID (Difference of Difference calculated as Within group LS mean difference of Active group - LS mean difference of Control group

^1^Calculated by regressing the within person difference in the outcome (pre and post treatment) on treatment group (active vs. delayed group). A negative value indicates a decline while a positive value indicates an increase post treatment

^2^ *p*–value for interaction by treatment (difference of the difference pre/post treatment per group)

**Fig. 4:**
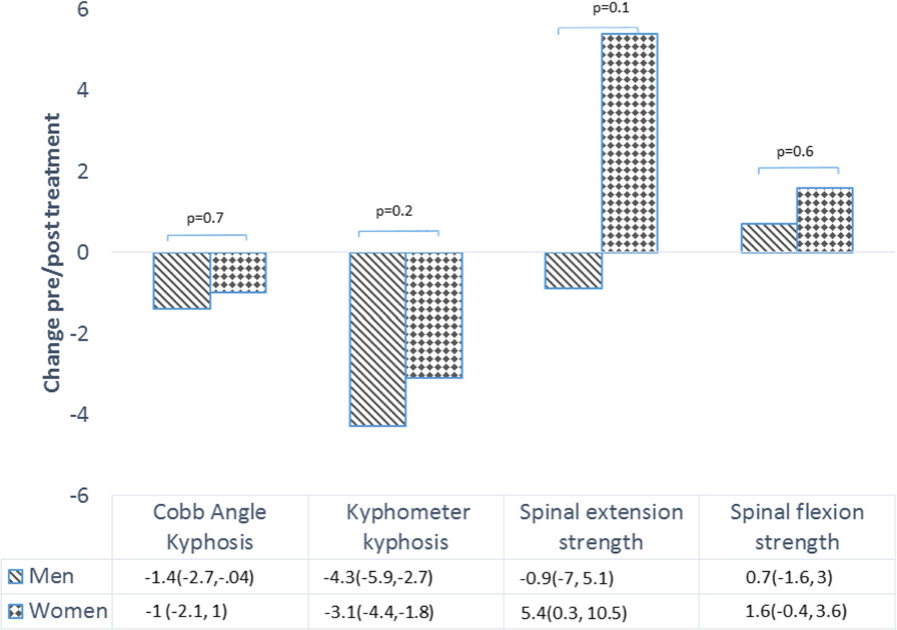
Sex Differences in Change in Kyphosis and Strength Outcomes After Exercise Intervention Negative change in Cobb angle and kyphosis and positive change in strength reflects improvement. Change pre/post treatment is reported with 95% confidence intervals; change in kyphosis is reported in degrees; change in strength is reported as peak torque normalized to body weight. *P* value indicates if the change differed significantly between men and women

There was no significant interaction in the treatment effect between men and women in the change in Cobb angle after both groups received the intervention, p = 0.7 (Fig. 4). In the pre-specified subgroup analyses, there was no significant interaction between DISH or prevalent vertebral fractures and change in Cobb angle, *p* > 0.1 (data not shown).

### Kyphometer-derived kyphosis, spinal strength, physical function, and HRQoL outcomes

Of the secondary outcomes, kyphometer-derived kyphosis and spinal flexion strength improved after the intervention. Participants in the active group had a 3.8 (95% CI: −5.3 to −2.3) degree decrease in kyphometer-derived kyphosis while those in the control group had a 1 (95% CI: −0.5 to 2.4) degree increase resulting in a difference-in-difference of 4.8 degrees and a *p*-value of 0.001 favoring the active group (Table 3). Participants in the active group had an 8 (95% CI: 2.9 to 13) percent increase in spinal extension peak torque adjusted for body weight while those in the control group had a 1.8 (95% CI: −3.2 to 6.8) percent increase in peak torque adjusted for body weight, resulting in a difference-in-difference of 6.2% and a *p*-value of 0.08 favoring the active group. Participants in the active group had a 2 (95% CI: 0.1 to 4) percent increase in spinal flexion peak torque adjusted for body weight while those in the control group had a 0.7 (95% CI:-2.6 to 1.3) percent decrease resulting in a difference-in-difference of 2.7% and a p-value of 0.05 favoring the active group. There were no significant between group differences in change in any physical function or HRQoL outcome, *p* > 0.05 (Table 3).

There were no significant sex differences in change in Cobb angle or kyphometer-derived kyphosis in response to the intervention, *p* > 0.1 (Fig. 4). The change in spinal extension peak torque was greater in women, with a 5.4 (95% CI: 0.3 to 10.5) percent increase in spinal extension peak torque adjusted for body weight versus a − 0.9 (95% CI: −7.0 to 5.1) percent decrease in men, however the difference between men and women was not significant (*p* = 0.1). There were no significant sex differences in change in physical function or HRQoL in response to the intervention, *p* > 0.1 (Table 4).

**Table 4:** Sex differences in change in physical function and HRQoL outcomes after exercise intervention

Outcome	Women (*n* = 60)	Men (*n* = 41)	*p*-value
	LS Mean change (95% CI)	LS Mean change (95% CI)	
Modified PPT (0–36 points)	−1.0 (−2.6 to 0.5)	−1.9 (−3.8 to 0.1)	0.51
4-m walk (meters/s)	0.02 (0 to 0.1)	0 (−0.1 to 0.1)	0.57
Timed Up and Go (seconds)	−0.1 (−0.4 to 0.1)	0.1 (−0.2 to 0.5)	0.22
Timed Loaded Standing (seconds)	3.2 (−4.3 to 10.6)	1.7 (−7.5 to 10.9)	0.81
Six Minute Walk Test (meters)	11.5 (− 3.3 to 26.3)	1.8 (−16.2 to 19.9)	0.41
SRS 30 Self-esteem (0–5 points)	0.25 (0.1 to 0.4)	0.2 (0.1 to 0.3)	0.52
Global health index (0–50)	0.7 (− 0.3 to 1.6)	0.5 (−0.6 to −1.6)	0.84
Physical function (t--score)	1.7 (0.3 to 3.2)	2.0 (0.3 to 3.8)	0.79

*p-value for interaction by sex with *p* < 0.1 indicating significance

### Adverse events

There were no serious adverse events (death, life-threatening adverse experiences, or related inpatient hospitalization) and no reportable adverse events associated with the study in either group according to federal regulations and UCSF Institutional Review board criteria. There were numerous non-reportable events in both groups, which included pain and stiffness felt in muscles several hours to days after testing or exercise, and resolved within an expected duration [43]. When we examined both groups during the 3-month period when they were allocated to the intervention, 95 (84.8%) reported a total of 129 different non-reportable events, with 10 falls. The majority of the musculoskeletal complaints (90%) were pre-existing and resolved within a couple of weeks. Twenty-seven control participants (49.1%) reported 31 different non-reportable events including 7 falls and 22 reports of musculoskeletal pain during the 3-month waitlist period.

## Discussion

In this randomized controlled waitlist trial of a kyphosis-specific exercise and posture training intervention, performed twice weekly for 3 months, we found that the magnitude of kyphosis improved in the clinical but not the radiographic measure of kyphosis. The clinical kyphometer measure of kyphosis progressed 1.0 (95% CI: −0.5 to 2.4) degree in the control group and was reduced by 3.8 (95% CI: −5.3 to −2.3) degrees in the active group in the initial 3 months, suggesting that this type of intervention may reduce hyperkyphosis and prevent its progression over 3 months.

We did not find a significant change in the primary outcome of change in radiographic Cobb angle of kyphosis that we previously reported after a similar kyphosis-specific exercise and posture training intervention over 6-months [25]. The between group difference of 1.7 degrees exceeded the 1.4 degree standard error of the measurement, but it was not statistically significant. Furthermore, the difference in change in Cobb angle did not reach the minimal detectable difference of 2.6 degrees. While we did find change in the clinical measure of kyphosis, a longer period of time may be needed before radiographic changes are established. It is also possible that differences in the superior landmarks used to assess kyphosis, the T23 interspace in the kyphometer-derived kyphosis, and the superior endplate of T4 in the radiographic Cobb angle of kyphosis, may confound these measurements because the T23 interspace may be more anterior than T4 in the sagittal plane.

The clinical response to the intervention is consistent with some but not all prior studies, and may be affected by the intensity and duration of the intervention. The 3.8 degree reduction in the clinical kyphometer measure of kyphosis that we observed is less than our prior uncontrolled pilot trial that reported a 6 degree reduction in kyphometer-derived kyphosis after a similar twice a week for 3 months exercise intervention in older women. In that trial, the resistance was progressed after every third study visit to maintain the resistance at a “hard to somewhat hard” level [24]. It is consistent with a similar intervention (SHEAF trial) that progressed the resistance according to the participant’s perception when the exercise was no longer “hard or somewhat hard” over a 3×/week for 6-month period [27]. The change in the clinical measure of kyphosis exceeded the Greendale, et al. trial [22] that reported a 3-degree improvement in kyphometer-derived kyphosis in the active group, albeit with no significant difference between the groups, *p* = 0.44, after a 3×/week for 6-month yoga randomized controlled trial with no strengthening component. The previous uncontrolled pilot with structured progression of resistance resulted in a 10.5% improvement over 3-months compared to a 7.3% improvement in our current trial with the same frequency and duration of study visits, but our current trial allowed participants to decide when to progress the resistance to maintain a level of “hard to somewhat hard”. Both of these trials resulted in a larger clinical change in kyphosis in 3-months than the changes reported in the 3×/week for 6-month SHEAF trial (6.7% change) and Greendale trial (5.2% change). Thus, it appears that 3-months may be sufficient to produce a clinical change in kyphosis, but a structured progression of resistance may produce better results over a shorter time. Future trials should include 10RM max testing at intervals throughout the trial to facilitate progression of the strengthening component of the intervention [44].

We also did not observe a change in either physical function or health-related quality of life measurements. In contrast to our pilot study that reported a significant improvement in the modified PPT, our cohort was quite robust at baseline with only 5 participants categorized in the mild frailty range. Moreover, these results are consistent with our previous SHEAF randomized controlled trial where the cohort was also quite robust [25]. Even a more frequent and longer intervention in SHEAF did not produce changes in physical function. Future trials may consider recruiting a more frail population, and including exercises that specifically target physical function. In the SHEAF trial, the SRS-30 self-image domain improved whereas we did not find a change in this outcome. It is possible that a longer term and more frequent intervention may be necessary to affect self-image.

There were no sex differences in the main treatment effects in our cohort despite sex differences in kyphosis, bone mineral density, spine strength, vertebral fractures and DISH at baseline. Interestingly, men in our cohort had better hip and spine bone mineral density than the women, yet they had more vertebral fractures. Men had more DISH which has previously been associated with vertebral fractures due to the ankylosis of vertebral segments that creates long lever arms that may increase risk of fracture even in low-energy injuries. DISH has also been associated with greater magnitude of kyphosis in older men and women, [45] and less progression of kyphosis in older women [16]. Regardless, participants in our cohort with DISH and vertebral fractures responded equally well to the intervention compared to those without DISH or prevalent vertebral fractures.

Men had greater spine strength at baseline, however there were no significant sex differences in their response to the intervention. Men did not improve in spinal extensor strength, whereas women increased spinal extensor peak torque by 5.4 (0.3, 10.5) percent, although the difference between men and women was not significant (*p* = 0.1). Previous study by DaBoit et al. [46] reported sex differences in change in knee extension peak torque after an 18-week resistance exercise training program in a cohort of older (>65 years) men and women. Maximal torque increased by 15.8% (SD = 10.6) in women and 41.7% (SD = 25.5) in men. In contrast, Lemmer et al. [47] found that changes in 1-RM strength in response to both strength training and detraining are affected by age, but not sex. Both older men and women increased knee extension muscle 1RM strength after 9-weeks of training. Our cohort increased spinal extension and flexion peak torque after the intervention in the active versus the control group, but the between group difference was not statistically significant, and there were no significant differences in spinal extension or flexion strength change between the men and women.

### Strengths/limitations

The strengths of our study are that it included 40% men and we investigated sex differences in response to a randomized controlled intervention, which adds to our understanding of sex-differences in response to a kyphosis-specific intervention. Also, the intervention was well tolerated with no reportable adverse events. However, the intervention was only 3 months in duration, and we did not test 10 RM maximum to control intensity, thus a longer term and more intense intervention may be needed to demonstrate changes in radiographic-measured kyphosis, physical function and HRQoL. In addition, our cohort was high functioning at baseline [34], and a cohort with a wider range of baseline functioning may be more reflective of the general population and also might be better able to demonstrate changes in physical function with the intervention. Furthermore, a greater proportion of the control group was classified with “mild frailty” on the modified PPT, even though there were no differences in the other physical function measures, and this may have influenced the results. Moreover, PPT was a secondary outcome, and we did not design the study to test for an interaction to determine if PPT was an effect modifier. Future studies are needed to investigate this type of intervention in lower functioning participants and whether this intervention prevents the progression of kyphosis that usually occurs over time.

## Conclusions

A targeted spine strengthening exercise and postural training program over 3 months had a non-statistically significant improvement in radiographic Cobb angle of kyphosis, *p* = 0.09, but improved the clinical kyphometer-measured kyphosis by 4.8 degrees in the active group compared to the control group. Results from our randomized controlled trial show that despite sex differences in baseline kyphosis and other musculoskeletal impairments that affect kyphosis, there were no sex differences in response to the intervention. For older men and women with hyperkyphosis, a recommendation for a spine strengthening exercise and posture training program may be considered.

## Abbreviations

≥: Greater than or equal
°: Degrees
BMD: Bone mineral density
DID: Difference of the difference
DISH: Diffuse idiopathic skeletal hyperostosis
HRQoL: Health-related quality of life
kg/m^2^: Kilogram/m^2^
SD: Standard deviation
SHEAF: Study of hyperkyphosis, exercise and function
SQ: Semi-quantitative Genant scoring
